# Supplementary material for: Tuneable reflexes control antennal positioning in flying hawkmoths
Source: Nat Commun. 2019 Dec 6;10:5593. doi: 10.1038/s41467-019-13595-3 (PMC6898381; doi:10.1038/s41467-019-13595-3)
Supplement: Supplementary file 1 — Supplementary Information [file 41467_2019_13595_MOESM1_ESM.pdf]

Supplementary Information

Tuneable reflexes control antennal positioning in flying hawkmoths

Natesan *et al.*

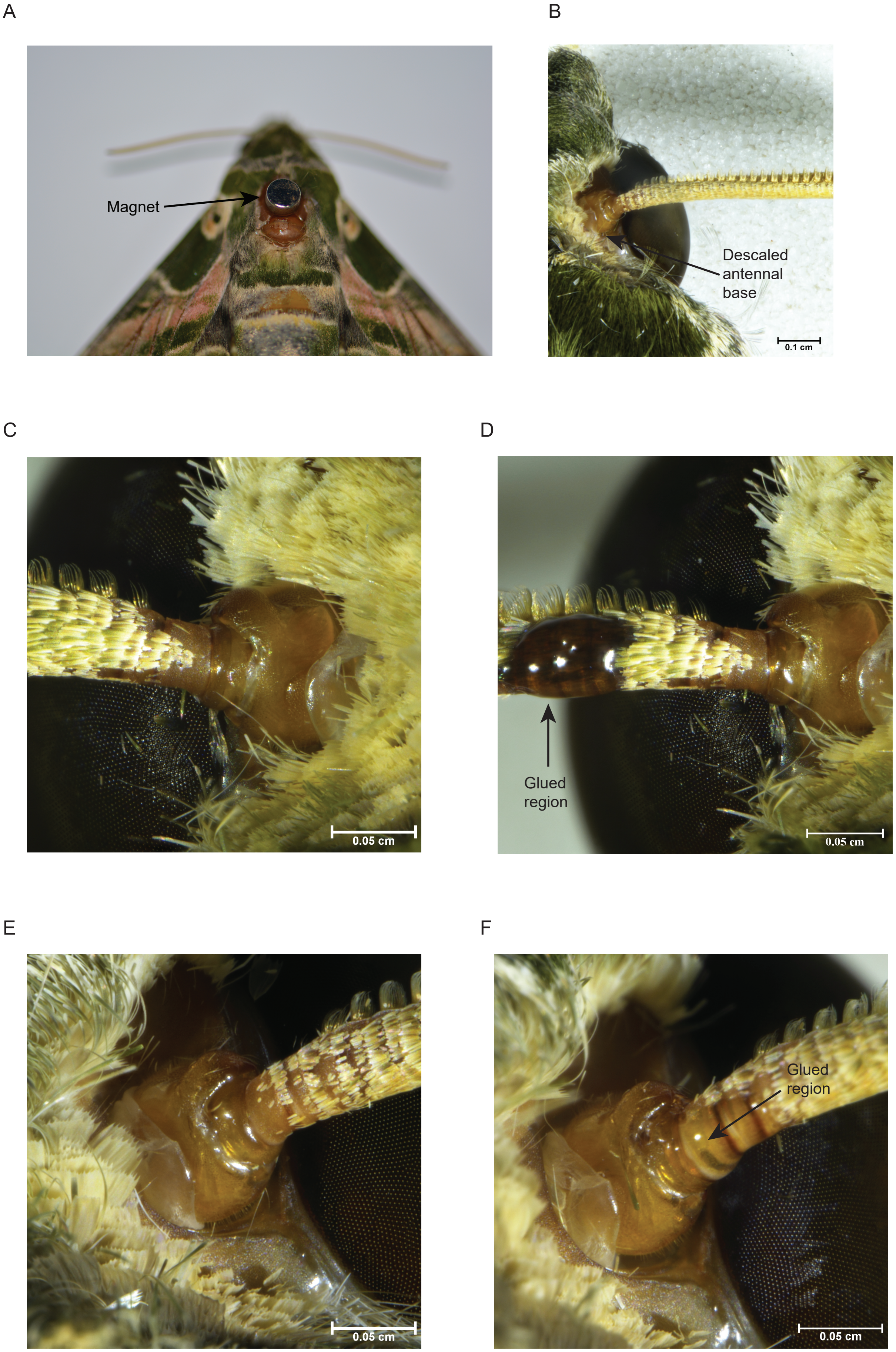

Supplementary Figure 1: Images of antennal manipulations

(A) Magnetic tether. The magnet was attached to the dorsal surface of the thorax. **(B)** Descaled antenna base. The antennae were carefully descaled, ensuring that none of the Böhm's bristles were damaged.
(C-D) Sham-treated moths. **(C)** Before and **(D)** after glue was applied to the third/fourth annulus.
(E-F) JO-restricted moths. **(E)** Before and **(F)** after the pedicel-flagellar joint was glued in order to restrict vibrations to the JO.

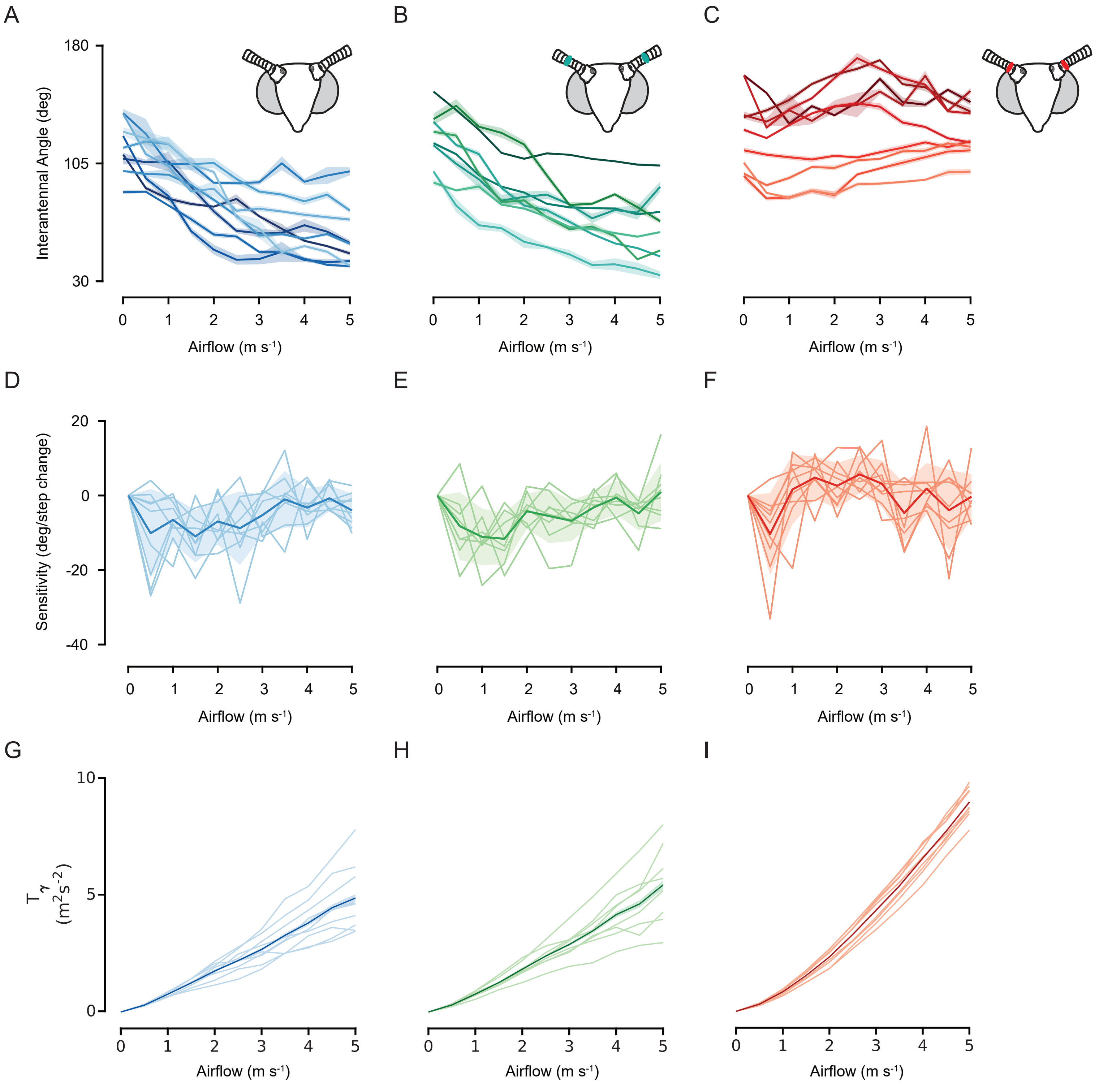

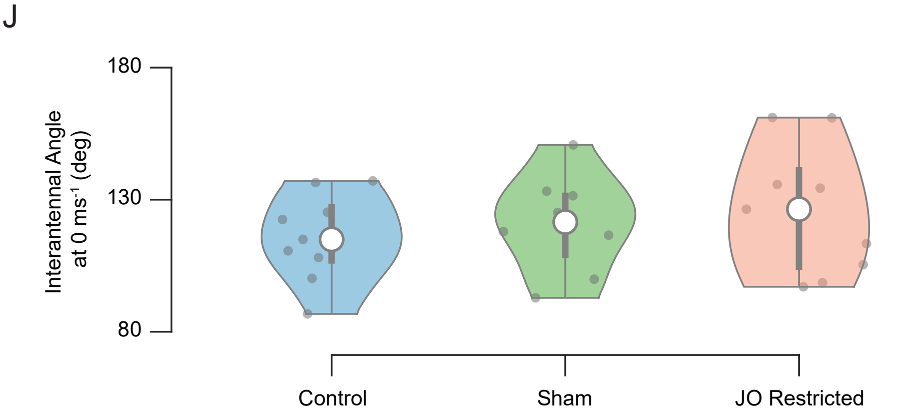

Supplementary Figure 2: Raw data for antennal positioning response.

(A-C) Antennal response of **(A)** Control, **(B)** Sham-treated moths and **(C)** JO-restricted moths. Raw data are shown here without baseline shifting.
(D-F) Sensitivity of antennal response to changes in airflow in **(D)** Control, **(E)** Sham-treated moths and **(F)** JO-restricted moths. The dark line represents the mean response of individual moths (individuals shown as lighter lines) and overlay represents the standard deviation. Sensitivity to airflow of control and sham-treated moths were correlated (ρ = 0.70) whereas sensitivity of JO restricted moths was not correlated with the other treatments or with changes in airflow (with control – ρ = -0.21; with sham – ρ = -0.09; with airflow – ρ = 0.04).
(G-I) Torque on the antennal base due to aerodynamic drag in **(G)** Control, **(H)** Sham-treated moths and **(I)** JO-restricted moths. Torque on the antennal base (T_γ) had non-linear dependence on antennal angle and airflow speed (equation derived from [30], see methods). Modulation of antennal angle in control and sham-treatment moths reduced the slope of T_γ for increases in airflow, in comparison to JO-restricted moths. However, T_γ increased with airflow for all three treatments (average slope for control: 45°, sham: 47°, JO-restricted: 62°. The slopes were from linear fits with adjusted $R^2 > 0.95$).
(J) Interantennal angle for no airflow (0 m s^{-1}) of all three treatments. The three treatments were not statistically different from each other (Kruskal Wallis test, p=0.78).

Supplementary Figure 3
Natesan, Saxena, Ekeberg and Sane

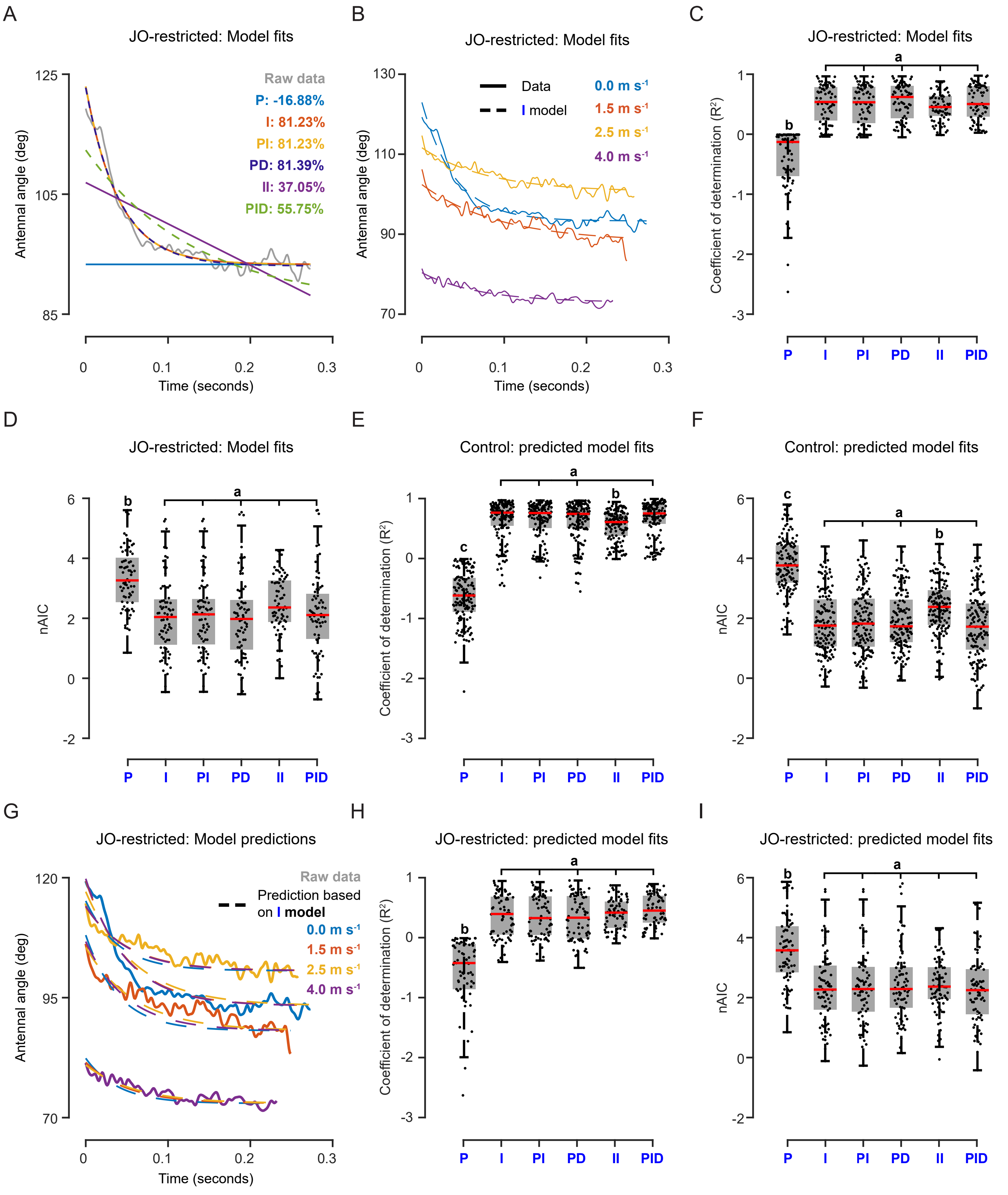

Supplementary Figure 3: Model fits and predictions of antennal response for control and JO-restricted moths.

(A-D) Model fits for the JO-restricted moth dataset. **(A)** Model fits for a representative trajectory. **(B)** Integral model fits for a representative dataset. **(C-D)** Goodness-of-fit for all the models (a, b represent statistically different groups, Kruskal Wallis, Nemenyi test, $p<0.01$; n=72 trajectories). The integral model (I) was the most parsimonious (median R^2 – P: -0.13, I: 0.54, PI: 0.53, PD: 0.62, II: 0.45, PID: 0.50; median nAIC - P: 3.26, I: 2.04, PI: 2.13, PD: 1.98, II: 2.36, PID: 2.11).
(E-F) Goodness-of-fit for control moth predictions. I, PI, PD, PID predicted the return trajectories (a, b, c represent statistically different groups, Kruskal Wallis, Nemenyi test, $p<0.01$; n=133 trajectories; median R^2 – P: -0.62, I: 0.76, PI: 0.76, PD: 0.74, II: 0.61, PID: 0.75; median nAIC - P: 3.76, I: 1.75, PI: 1.82, PD: 1.73, II: 2.38, PID: 1.72).
(G-I) Model predictions for JO-restricted moth dataset. Integral model predictions for **(G)** a representative dataset. **(H-I)** Goodness-of-fit for predictions for all the models (a, b represent statistically different groups, Kruskal Wallis, Nemenyi test, $p<0.01$; n=72 trajectories; median R^2 – P: -0.42, I: 0.39, PI: 0.33, PD: 0.33, II: 0.42, PID: 0.45; median nAIC - P: 3.58, I: 2.27, PI: 2.28, PD: 2.29, II: 2.37, PID: 2.25).

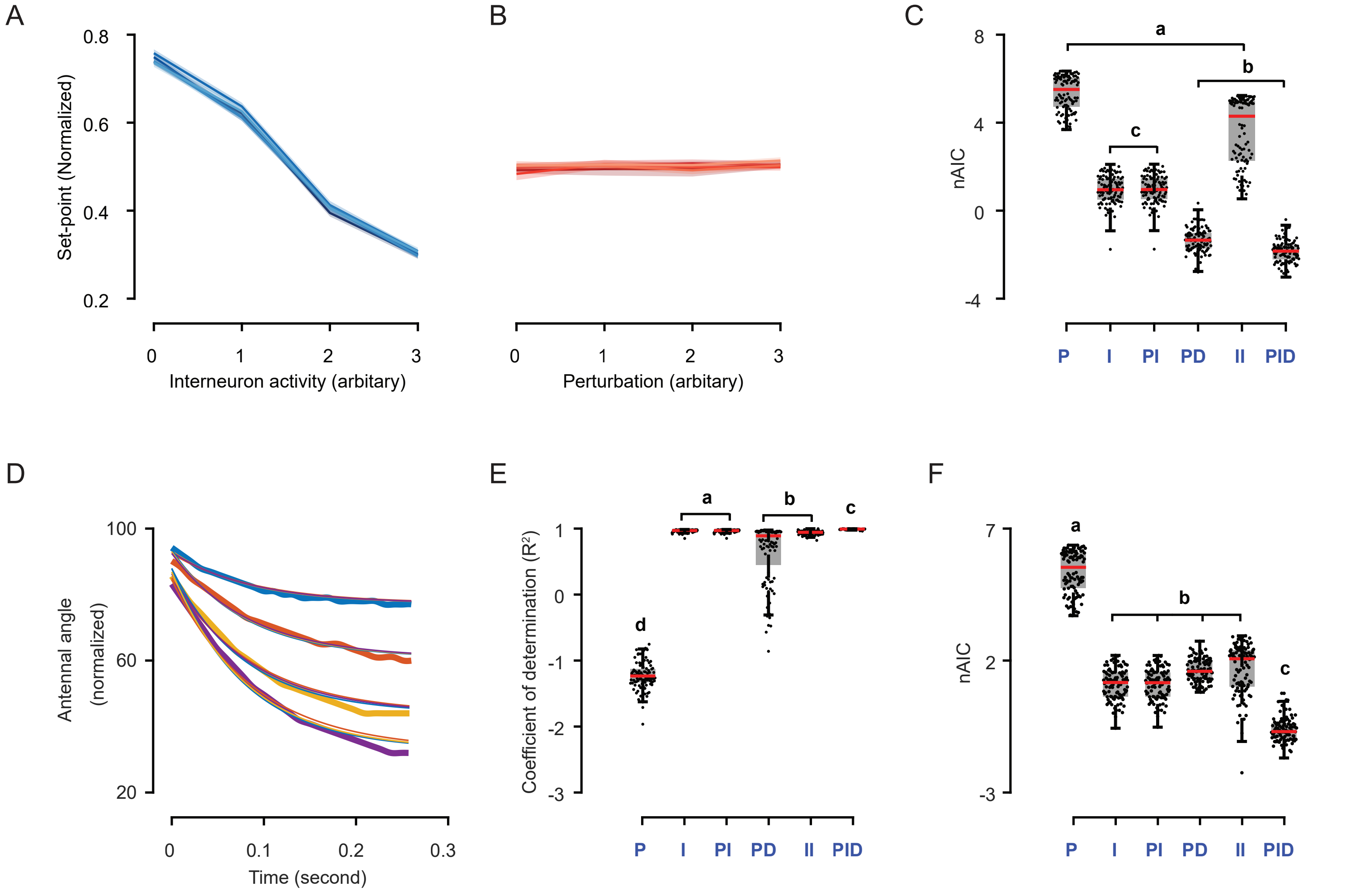

Supplementary Figure 4: Integral model predictions for neural circuit simulation.

(A-B) Set-points of neural circuit simulation. Interneuron activity **(A)** modulated the set-point of the simulated neural circuit, which was **(B)** robustly kept constant regardless of the amplitude of perturbations.
(C) Model fits for neural circuit simulation. Goodness-of-fit for model predictions for all the models (Kruskal Wallis, Nemenyi test, $p<0.01$; n=100 trajectories; median nAIC - P: 5.51, I: 0.95, PI: 0.96, PD: -1.34, II: 4.29, PID: -1.84).
(D-F) Model predictions for neural circuit simulation. **(D)** Integral model predictions for a representative dataset from the simulated neural circuit. **(E-F)** Goodness-of-fit for model predictions for all the models (Kruskal Wallis, Nemenyi test, $p<0.01$; n=100 trajectories; median R^2 – P: -1.23, I: 0.97, PI: 0.97, PD: 0.94, II: 0.94, PID: 1.00; median nAIC - P: 5.50, I: 1.16, PI: 1.16, PD: 1.71, II: 2.05, PID: -0.81).

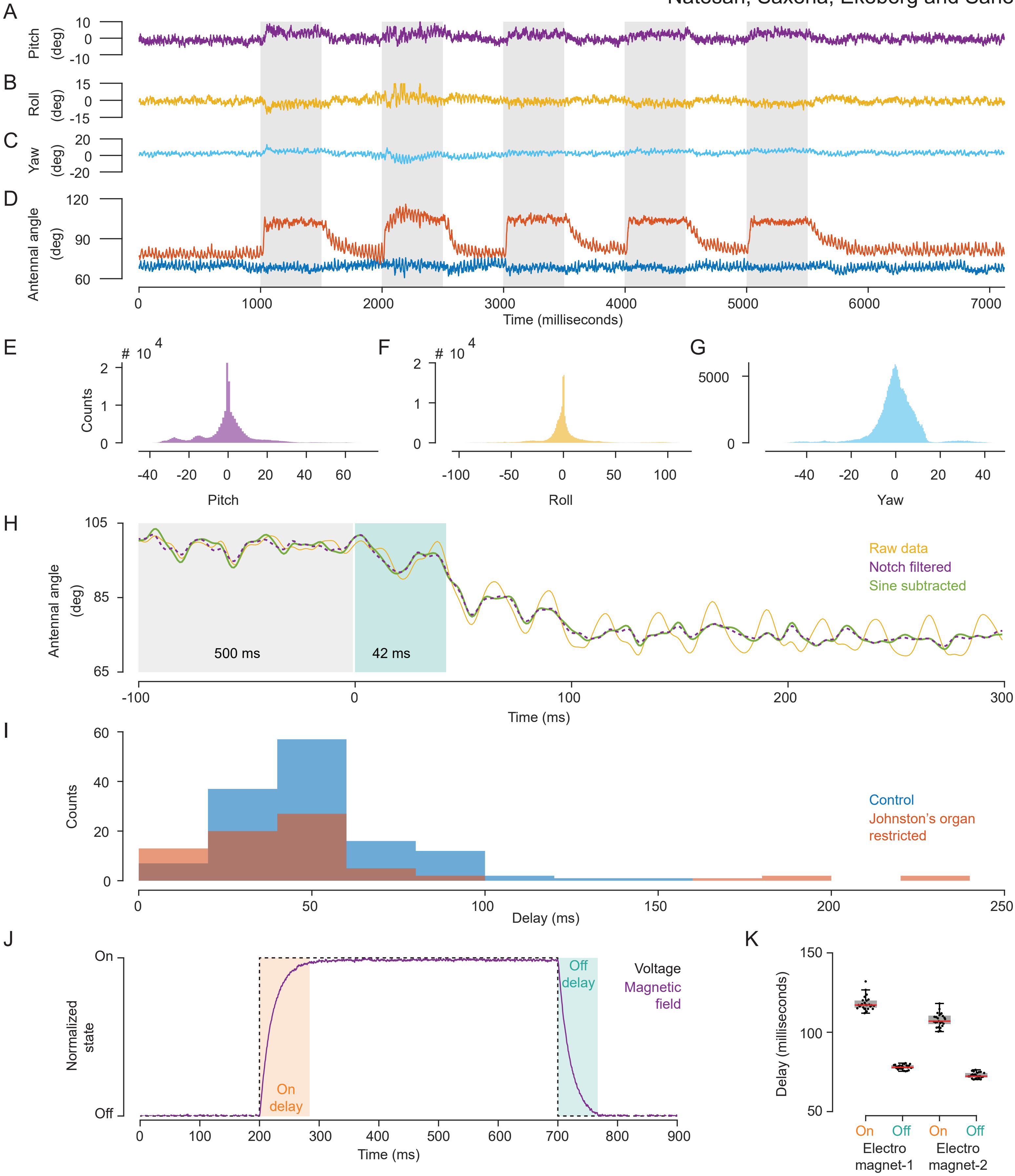

Supplementary Figure 5: Head rotations and electromagnet release delays

(A-G) Head rotations during electromagnet perturbations. **(A)** Pitch, **(B)** Roll and **(C)** Yaw of the head. Grey boxes indicate electromagnet "on". The moth rotated its head slightly in response to left antenna perturbation, but because of the head-centric-system, **(D)** the right antennal angle (internal control, blue) did not change. The left antennal angle (red) changed as expected. Distributions of **(E)** Pitch, **(F)** Roll and **(G)** Yaw in all experiments.
(H-I) Filtering of raw antennal angle and estimating release point. **(H)** Raw antennal angle (yellow) was filtered by estimating and subtracting wing beat frequencies [green, see methods, akin to using a notch filter (dotted blue line)]. Release point was estimated as point where the antenna crossed 25% of the difference between perturbed sand final settled angles (set-point). Delay depended on many factors: distance of electromagnet, differences in antennal inertia, etc. and was **(I)** centred around 50 ms for both control and JO-restricted moths.
(J-K) Characterization of the electromagnet. On-off delay was characterized by measuring the magnetic fields using a Hall effect sensor (DRV5053). The electromagnet was placed 2 cm from the sensor, roughly the same distance as for the antenna. **(J)** Normalized voltage inputs to the electromagnet (black) and measured voltage output from Hall effect sensor (purple). On- (red overlay) and off-delay (blue overlay) were computed as time taken for magnetic fields to stabilize to 99% CI of the mean on and off voltages, respectively. **(K)** Box plot of on- and off-delays of both electromagnets used (median: E1-117.1 ms on, 77.0 ms off; E2-107 ms on, 72.3 ms off; overall median: 113.3 ms on, 76.1 ms off).

Supplementary Figure 6
Natesan, Saxena, Ekeberg and Sane

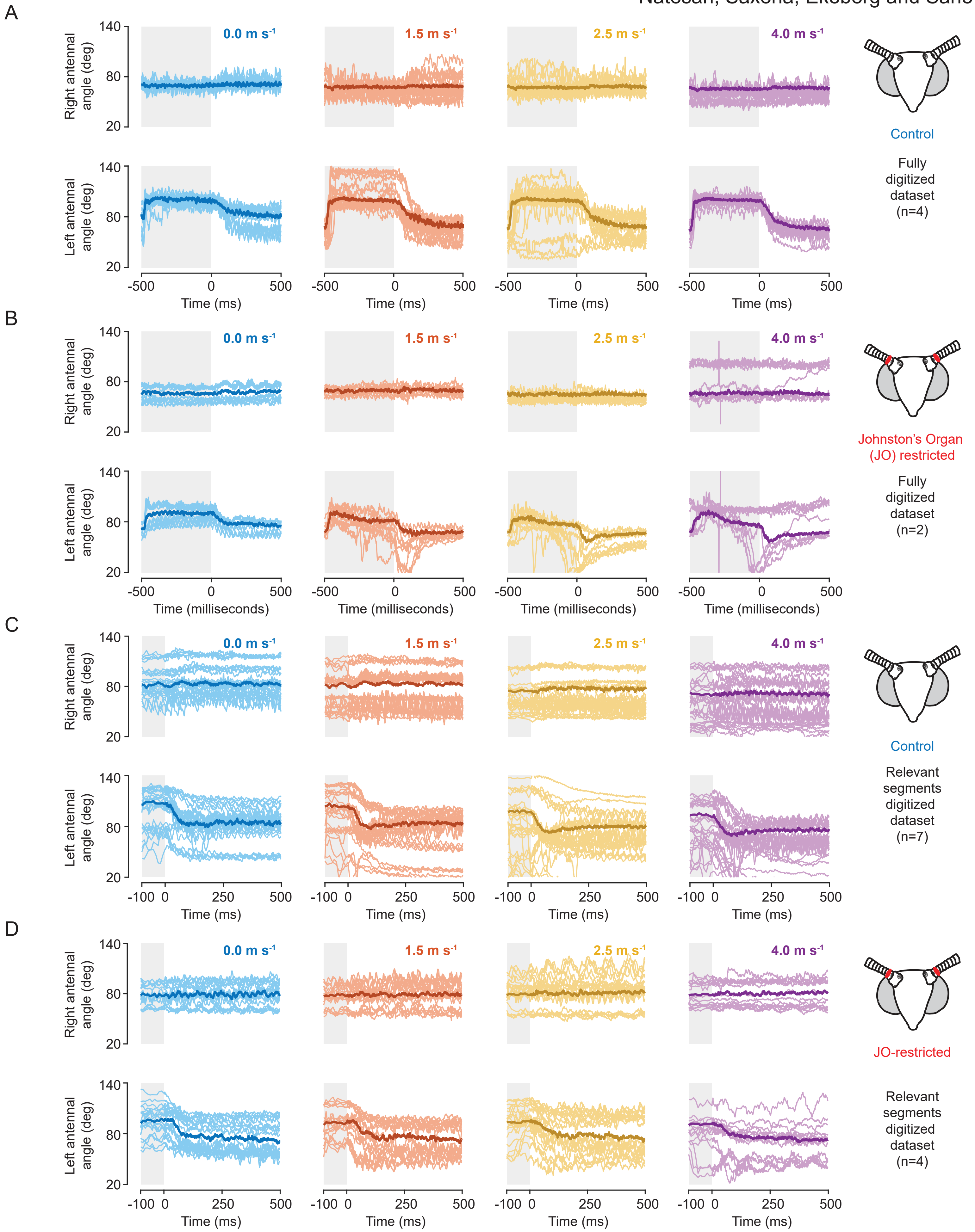

Supplementary Figure 6: Raw data traces of antennal response to perturbation in control and JO-restricted moths.

(A-B) Fully digitized antennal response to perturbation of Control (n=4) and JO-restricted (n=2) moth trials. The colour indicates airflow value, with the light traces representing individual trajectories and the dark trace representing the average response. Note that the right antennal angle is typically constant and unaffected by perturbation of the left antenna.
(C-D) Raw antennal response to perturbation of the remaining data set [Control (n=7) and JO-restricted (n=4)]. In the rest of the dataset, only frames 100 ms before and 500 ms after perturbation was digitized. This was sufficient to characterize the response of the antennal positioning reflex (see Methods for more information). Raw traces for all trajectories in control and JO restricted moths digitized in this manner are shown here.
