# Supplementary material for: Tuneable reflexes control antennal positioning in flying hawkmoths
Source: Nat Commun. 2019 Dec 6;10:5593. doi: 10.1038/s41467-019-13595-3 (PMC6898381; doi:10.1038/s41467-019-13595-3)
Supplement: Supplementary file 3 — Reporting Summary [file 41467_2019_13595_MOESM3_ESM.pdf]

nature research

Corresponding author(s): Sanjay P. Sane
Last updated by author(s): Nov 5, 2019

Reporting Summary

Nature Research wishes to improve the reproducibility of the work that we publish. This form provides structure for consistency and transparency in reporting. For further information on Nature Research policies, see Authors & Referees and the Editorial Policy Checklist .

Statistics

For all statistical analyses, confirm that the following items are present in the figure legend, table legend, main text, or Methods section.

n/a	Confirmed	
☐	☒	The exact sample size (*n*) for each experimental group/condition, given as a discrete number and unit of measurement
☐	☒	A statement on whether measurements were taken from distinct samples or whether the same sample was measured repeatedly
☐	☒	The statistical test(s) used AND whether they are one- or two-sided<br>*Only common tests should be described solely by name; describe more complex techniques in the Methods section.*
☒	☐	A description of all covariates tested
☐	☒	A description of any assumptions or corrections, such as tests of normality and adjustment for multiple comparisons
☐	☒	A full description of the statistical parameters including central tendency (e.g. means) or other basic estimates (e.g. regression coefficient) AND variation (e.g. standard deviation) or associated estimates of uncertainty (e.g. confidence intervals)
☐	☒	For null hypothesis testing, the test statistic (e.g. *F*, *t*, *r*) with confidence intervals, effect sizes, degrees of freedom and *P* value noted<br>*Give P values as exact values whenever suitable.*
☒	☐	For Bayesian analysis, information on the choice of priors and Markov chain Monte Carlo settings
☒	☐	For hierarchical and complex designs, identification of the appropriate level for tests and full reporting of outcomes
☒	☐	Estimates of effect sizes (e.g. Cohen's *d*, Pearson's *r*), indicating how they were calculated

Our web collection on statistics for biologists contains articles on many of the points above.

Software and code

Policy information about availability of computer code

Data collection	Autotracker codes used for digitization are available in Github and Zenodo with an identifier of “10.5281/zenodo.3517748”
Data analysis	Analysis codes are available in Github and Zenodo with a unique identifier of “doi:10.5281/zenodo.3515775”.

For manuscripts utilizing custom algorithms or software that are central to the research but not yet described in published literature, software must be made available to editors/reviewers. We strongly encourage code deposition in a community repository (e.g. GitHub). See the Nature Research guidelines for submitting code & software for further information.

Data

Policy information about availability of data

All manuscripts must include a data availability statement. This statement should provide the following information, where applicable:

- Accession codes, unique identifiers, or web links for publicly available datasets
- A list of figures that have associated raw data
- A description of any restrictions on data availability

The data that support the findings of this study are available in Zenodo with a unique identifier of “doi:10.5281/zenodo.3515753”. Raw experiment videos from which the data was digitized are available upon request from the corresponding author.

Field-specific reporting

Please select the one below that is the best fit for your research. If you are not sure, read the appropriate sections before making your selection.

☒ Life sciences ☐ Behavioural & social sciences ☐ Ecological, evolutionary & environmental sciences

Life sciences study design

All studies must disclose on these points even when the disclosure is negative.

Sample size	Sample sizes (individuals) for the airflow-dependent antennal positioning experiment (Fig 1) - Control moths: n=9; Sham-treated moths: n=8; JO-restricted: n=9<br>Sample sizes for the antennal perturbations for different airflow (Fig 2) - Control moths: n=11 trials from 9 individuals; JO-restricted: n=6 trials from 6 individuals.<br>Sample sizes for neural circuit simulations (Fig 5) - Intrinsic set-point: n=100 trajectories (trials, random seed); airflow-dependent set-point: n=100 trajectories (trials, random seed)
Data exclusions	All experiments: airflow-dependent antennal positioning in hawkmoths was recorded during one flight bout. In cases where this happened in multiple bouts, the experiments were not digitized. This is because antennal positioning in hawkmoths occurs both in preparation for and during flight (Dorsett, 1962).<br>JO-restricted experiments: The restriction of the pedicel-flagellar joint was checked under the microscope before and after experiments in JO-restricted moths. If the glue had cracked, the experiments were discarded due to unreliable restriction of the pedicel-flagellar joint.
Replication	Both airflow-dependent antennal positioning and role of JO in sensing airflow and controlling antennal positioning were first studied in a pilot dataset (n = 5). Once the preliminary analysis confirmed the experiment, it was formalized and replicated.
Randomization	All moths were randomly picked from a one-day old batch of moths. 3-6 experiments comprising of 1-2 individuals per treatment were performed. Each moth was alloted a number and the details of the treatment were documented in a datasheet.
Blinding	Blinding is not applicable here, as we recorded and quantifed the effect of a particular treatment on the antennal response.

Reporting for specific materials, systems and methods

We require information from authors about some types of materials, experimental systems and methods used in many studies. Here, indicate whether each material, system or method listed is relevant to your study. If you are not sure if a list item applies to your research, read the appropriate section before selecting a response.

Materials & experimental systems

n/a	Involved in the study
☒	☐ Antibodies
☒	☐ Eukaryotic cell lines
☒	☐ Palaeontology
☒	☐ Animals and other organisms
☒	☐ Human research participants
☒	☐ Clinical data

Methods

n/a	Involved in the study
☒	☐ ChIP-seq
☒	☐ Flow cytometry
☒	☐ MRI-based neuroimaging
