# Supplementary material for: Tuneable reflexes control antennal positioning in flying hawkmoths
Source: Nat Commun. 2019 Dec 6;10:5593. doi: 10.1038/s41467-019-13595-3 (PMC6898381; doi:10.1038/s41467-019-13595-3)
Supplement: Supplementary file 4 — Description of Additional Supplementary Files [file 41467_2019_13595_MOESM4_ESM.pdf]

Description of Additional Supplementary Files

File Name: Supplementary Movie 1
Description: **Antennal response to increasing airflow in Control moths.** This movie shows the antennal positioning response of a Control moth to increasing airflow. Data from 11 different airflow speeds (0 to 5 m s-1 in steps of 0.5 m s-1) are shown here. Individual videos have been down sampled by 10 and stitched together. The changes in airflow are shown in the top left corner of the screen. The antennal tips are indicated using red open circles and previous locations of the antenna are shown by closed red diamonds.

File Name: Supplementary Movie 2
Description: **Antennal response to increasing airflow in Sham-treated moths.** This movie shows the antennal positioning response of a Sham-treated moth to increasing airflow. Pooling of videos and representation of antennal tips is same as Supplementary Movie 1.

File Name: Supplementary Movie 3
Description: **Antennal response to increasing airflow in JO-restricted moths.** This movie shows the antennal positioning response of a JO-restricted moth to increasing airflow. Pooling of videos and representation of antennal tips is same as Supplementary Movie 1.

File Name: Supplementary Movie 4
Description: **Antennal response to perturbations for different airflows in Control moths.** This movie shows the antennal response to perturbations of a Control moth at different airflow. Data from four different airflow speeds (0, 1.5, 2.5 and 4 m s-1) are shown here. Individual videos have been down sampled and trimmed to show only three perturbation steps per airflow. The changes in airflow are shown in the bottom right corner of the screen. The antennal tips are indicated are shown by closed cyan diamonds when the electromagnet is on (also indicated by an LED on), and closed red diamonds with the electromagnet is off

File Name: Supplementary Movie 5
Description: **Antennal response to perturbations for different airflows in JO-restricted moths.** This movie shows the antennal response to perturbations of a JO-restricted moth at different airflow. Pooling of videos and representation of antennal tips is same as Supplementary Movie 4.
